# Exposure to human relevant mixtures of halogenated persistent organic pollutants (POPs) alters neurodevelopmental processes in human neural stem cells undergoing differentiation

**DOI:** 10.1016/j.reprotox.2020.12.013

**Published:** 2021-03

**Authors:** Nichlas Davidsen, Anna Jacobsen Lauvås, Oddvar Myhre, Erik Ropstad, Donatella Carpi, Emilio Mendoza-de Gyves, Hanne Friis Berntsen, Hubert Dirven, Ragnhild E Paulsen, Anna Bal-Price, Francesca Pistollato

**Affiliations:** a Department of Environmental Health, Section for Toxicology and Risk Assessment, Norwegian Institute of Public Health, Oslo, Norway; b Department of Production Animal Clinical Sciences, Faculty of Veterinary Medicine, Norwegian University of Life Sciences, Oslo, Norway; c European Commission, Joint Research Centre (JRC), Ispra, Italy; d National Institute of Occupational Health, Oslo, Norway; e Section for Pharmacology and Pharmaceutical Biosciences, Department of Pharmacy, University of Oslo, Norway

**Keywords:** Persistent organic pollutants, Developmental neurotoxicity, Human neural stem cells, Neurite outgrowth, Synaptogenesis, Aryl hydrocarbon receptor, Mathematical modelling

## Abstract

Halogenated persistent organic pollutants (POPs) like perfluorinated alkylated substances (PFASs), brominated flame retardants (BFRs), organochlorine pesticides and polychlorinated biphenyls (PCBs) are known to cause cancer, immunotoxicity, neurotoxicity and interfere with reproduction and development. Concerns have been raised about the impact of POPs upon brain development and possibly neurodevelopmental disorders. The developing brain is a particularly vulnerable organ due to dynamic and complex neurodevelopmental processes occurring early in life. However, very few studies have reported on the effects of POP mixtures at human relevant exposures, and their impact on key neurodevelopmental processes using human *in vitro* test systems. Aiming to reduce this knowledge gap, we exposed mixed neuronal/glial cultures differentiated from neural stem cells (NSCs) derived from human induced pluripotent stem cells (hiPSCs) to reconstructed mixtures of 29 different POPs using concentrations comparable to Scandinavian human blood levels. Effects of the POP mixtures on neuronal proliferation, differentiation and synaptogenesis were evaluated using *in vitro* assays anchored to common key events identified in the existing developmental neurotoxicity (DNT) adverse outcome pathways (AOPs). The present study showed that mixtures of POPs (in particular brominated and chlorinated compounds) at human relevant concentrations increased proliferation of NSCs and decreased synapse number. Based on a mathematical modelling, synaptogenesis and neurite outgrowth seem to be the most sensitive DNT *in vitro* endpoints. Our results indicate that prenatal exposure to POPs may affect human brain development, potentially contributing to recently observed learning and memory deficits in children.

## Highlights

• Mixtures of 29 halogenated POPs were reconstructed based on human blood levels.
• *In vitro* assays permitted to evaluate DNT effects in human iPSC-derived NSCs.
• POPs at human blood levels increased proliferating NSCs and decreased synapses.
• Mathematical modelling showed that synaptogenesis was the most sensitive DNT endpoint.
• Data support a possible link between POPs and neurodevelopmental disorders.

## 1 Introduction

Halogenated persistent organic pollutants (POPs) are long-lived compounds that are present in our daily environment and of concern to human health [1]. The toxic properties of POPs are well documented and many are classified as carcinogenic (e.g., 2,3,7,8-tetrachlorodibenzo-p-dioxin (TCDD) and polychlorinated biphenyls (PCBs) [2], polybrominated diphenyl ethers (PBDEs) [3], immunotoxic (e.g., perfluoroalkyl substances (PFASs) [4], or neurotoxic (e.g., BDE-47 [5], and may affect reproduction and development (e.g., PCB-118 [6], endosulfan [7]). Brominated and chlorinated POPs are hydrophobic [8] and accumulate mainly in lipid rich tissues, while perfluorinated compounds tend to show a higher affinity for plasma proteins [9]. Halogenated POPs have been shown to pass the blood brain barrier (BBB) [[10], [11], [12]], and accumulate in brain tissue without being metabolised, due to long biological half-lives [8,13]. Species differences in serum half-lives have been reported for a number of POPs, making it difficult to interpret animal data as a basis for human hazard assessment. Due to their persistent behaviour in the environment, bio-accumulative and toxic properties, some POPs have been banned or are nominated to be banned from production via the Stockholm Convention, and those still on the market are regulated through Registration, Evaluation and Authorization of Chemicals (REACH). However, many compounds have not been tested for toxic properties, and banned POPs are still found in both human and animal tissues [14].

Epidemiological studies report that various POPs are present in the blood of children, as well as in breast milk [15,16]. Additionally, POPs have been shown to cross the placenta barrier [17], possibly reaching the developing nervous system of the foetus through an immature BBB [18]. Moreover, depending on the ability of different POPs to cross the placenta, there may be differences in POPs distribution in foetal and maternal blood [19,20].

The developing brain undergoes complex and specific developmental processes, such as the proliferation of neural stem cells (NSCs), commitment of neuronal and glial progenitor cells, followed by migration (occurring after 8 postconceptional weeks, PCW), differentiation into various neuronal and glial subtypes, synaptogenesis (starting after 12–13 PCW), pruning (occurring after birth), myelination (taking place after 24 PCW), networking and terminal functional neuronal and glial maturation [[21], [22], [23], [24], [25], [26]].

From animal studies, it is well documented that during critical periods of brain development, even low exposures to environmental chemicals, which rarely affect adults, can disrupt brain development and maturation [21,27], potentially leading to adverse effects. This suggests that the nervous system of the developing foetus is particularly vulnerable to POP mediated interference, which may contribute to the recently observed increase in neurodevelopmental disorders [[28], [29], [30], [31], [32]], including learning disabilities, autism spectrum disorders (ASD) and attention-deficit hyperactivity disorder (ADHD) in children [33,34]. However, it is difficult to prove causality between adverse effects and specific groups of chemicals in epidemiological studies, since humans are continuously exposed to a complex mixture of various classes of chemicals.

Traditional toxicity testing is based on assessment of single compounds at relatively high doses, although human exposure more closely resembles that of chemicals in complex mixtures at very low levels and long-term exposure. These complex mixtures may interact in an additive, synergistic or antagonistic manner, triggering alteration of DNT endpoints [35], which to a certain extent can be predicted by modelling approaches.

According to the new toxicity testing paradigm, it is crucial to identify the molecular/cellular and tissue mechanisms/pathways underlying a toxic effect and the relationship between identified key events (KEs) resulting in a specific adverse outcome (AO). These mechanisms should, if possible, be investigated in human relevant test systems [36].

Previously, we reconstructed an environmentally relevant mixture of POPs for use in animal and *in vitro* experimental studies, containing 29 different chlorinated, brominated, and perfluorinated compounds [37]. Included compounds were not selected based on a specific mechanism of action but largely on a literature review on POPs found in food, blood and breast milk in Scandinavia, and on their listing as POPs under the Stockholm Convention on Persistent Organic Pollutants (SCPOP) [38]. The concentrations for the *in vitro* experiments were set to mimic the levels found in human blood, maintaining the proportions of the different compounds [37]. We have recently shown that this POP mixture and/or its single congeners inhibit the transactivation activity of the aryl hydrocarbon receptor (AhR) in three transgenic cell lines [39], and induce NMDA receptor mediated excitotoxicity in rat cerebellar granule neurons [40].

AhR, which controls the transcription of xenobiotic metabolizing enzymes, has also been shown to regulate neuronal differentiation in different models, modulating dendritic morphology [[41], [42], [43]], hippocampal neurogenesis and functions [44], cerebellar granule neuronal precursor cell number [45], poststroke astrogliosis and neurogenesis [46]. Moreover, AhR activation has been shown to occur in response to POPs in murine (but not human) neural progenitor cells, due to species-specific differences in AhR expression [47]. Along the same line, AhR protein has not been detected in human foetal brains from the second trimester of pregnancy [48], suggesting that AhR signalling may not be active in undifferentiated neural precursors.

In rat cerebellar granule neurons, the toxicity of perfluorinated compounds increased with carbon chain length, and for molecules with a similar chain length, a sulfonate functional group led to greater toxicity than a carboxyl group [49]. We have recently shown that maternal exposure to the POP mixture during pregnancy and weaning resulted in offspring hippocampal gene expression changes related to brain function and learning and memory deficits in mice offspring tested in the Barnes maze. Most compounds detected in dams were also found in offspring brain samples, indicating transfer of these compounds across the placenta as well as the BBB [50].

The aim of this study was to investigate through an *in vitro* and mathematical modelling approach, whether exposure to this complex mixture of POPs at human relevant concentrations affects key neurodevelopmental processes that could contribute to the POP-induced effects observed in animal and human studies. To achieve this, we used NSC-derived mixed neuronal/glial cultures originally obtained from human induced pluripotent stem cells (hiPSCs), and exposed them during the differentiation process to different combinations of POP mixtures at concentrations found in Scandinavian human blood (1x), one lower concentration (0.5 times human blood), and four higher concentrations (10, 100, 500 and 1000 times human blood levels).

## 2 Materials and methods

### 2.1 POP mixtures preparation

The mixtures of POPs were designed and prepared at the Norwegian University of Life Sciences as described in Berntsen et al. (2017) [37]. The concentrations tested were based on levels found in blood of people living in Scandinavia. The stocks used in the present study had a concentration of 10^6^ times human blood levels and were diluted down to relevant concentrations for use in the different experiments, using DMSO as a solvent. Most of the chlorinated and brominated compounds included in the mixture are banned from use and production under the SCPOP with certain exemptions, such as e.g. the use of *p,p’*-DDE as an antimalarial agent. Of the perfluorinated compounds, perfluorooctane sulfonate (PFOS) and perfluorooctanoic acid (PFOA) are listed under the SCPOP with exemptions, whereas perfluorohexanesulfonate (PFHxS) is proposed for listing [38]. Perfluorononanoic acid (PFNA), perfluorodecanoic acid (PFDA), and perfluoroundecanoic acid (PFUnDA), not yet on the SCPOP list, were also included in the mixture due to reported long elimination half-lives in humans [51,52]. We have previously shown that these chemicals as single compounds adversely affect cell viability in cultures of rat cerebellar granule neurons [49].

Seven POP mixtures were created using the compounds and the concentrations shown in Table 1. In particular, a total mixture contained: PFHxS, PFOS, PFOA, PFNA, PFDA, and PFUnDA, referred to as *‘PerF’*; brominated diphenyl ethers (BDE-47, BDE-99, BDE-100, BDE-153, BDE-154, BDE-209), and hexabromocyclododecane (HBCD), referred to as *‘Br’*; polychlorinated biphenyls (PCB 28, PCB 52, PCB 101, PCB 118, PCB 138, PCB 153, PCB 180), p,p'-DDE, HCB, α-chlordane, oxychlordane, *trans*-nonachlor, α-HCH, β-HCH, γ-HCH and dieldrin, referred to as ‘*Cl*’ (hereafter named as ‘*PerF + Br + Cl’*). Six additional mixtures containing ‘*PerF + Br’*, ‘*PerF + Cl’*, ‘*Br + Cl’*, and ‘*PerF’*, ‘*Br’*, or ‘*Cl’* compounds alone were created. The sub-mixtures were made to enable the assessment of effects upon adding or removing one or more chemical groups on different endpoints. Sources, purities and CAS numbers of the compounds included in the mixtures are presented in Supplementary Table 1. Chemical levels in the seven mixtures were measured and verified as described in [37]. Working stocks were prepared by creating suitable dilutions in DMSO in glass vials at concentrations 1000 times higher than the final exposure concentrations in the medium of 0.5x, 1x, 10x, 100x, 500x and 1000x, where x indicates ‘times human blood concentrations’ (Table 1). Vials were stored in −80 °C and fresh dilutions in medium were prepared for each medium change.

**Table 1:** Exposure concentrations of POP mixtures in the medium.

	Exposure concentrations - times human blood levels											
Compounds	0.5x	1x	10x	100x	500x	1000x	0.5x	1x	10x	100x	500x	1000x
	nM						ng/mL					
**Chlorinated compounds**												
PCB 28	0.016	0.031	0.311	3.11	15.6	31.1	0.004	0.008	0.08	0.8	4	8
PCB 52	0.010	0.021	0.205	2.05	10.3	20.5	0.003	0.006	0.06	0.6	3	6
PCB 101	0.012	0.025	0.245	2.45	12.3	24.5	0.004	0.008	0.08	0.8	4	8
PCB 118	0.069	0.138	1.379	13.79	69.0	137.9	0.023	0.045	0.45	4.5	23	45
PCB 138	0.215	0.430	4.295	42.95	214.8	429.5	0.078	0.155	1.55	15.5	78	155
PCB 153	0.349	0.698	6.983	69.83	349.2	698.3	0.126	0.252	2.52	25.2	126	252
PCB 180	0.170	0.339	3.39	33.90	169.5	339.0	0.067	0.134	1.34	13.4	67	134
*p,p'*-DDE	0.533	1.066	10.659	106.59	533.0	1065.9	0.170	0.339	3.39	33.9	170	339
HCB	0.114	0.228	2.282	22.82	114.1	228.2	0.033	0.065	0.65	6.5	33	65
α-chlordane	0.012	0.024	0.237	2.37	11.9	23.7	0.005	0.010	0.1	1	5	10
oxychlordane	0.017	0.033	0.33	3.30	16.5	33.0	0.007	0.014	0.14	1.4	7	14
*trans*-nonachlor	0.050	0.099	0.991	9.91	49.6	99.1	0.022	0.044	0.44	4.4	22	44
α-HCH	0.008	0.017	0.168	1.68	8.4	16.8	0.003	0.005	0.05	0.5	3	5
β-HCH	0.038	0.076	0.756	7.56	37.8	75.6	0.011	0.022	0.22	2.2	11	22
γ-HCH	0.008	0.017	0.168	1.68	8.4	16.8	0.003	0.005	0.05	0.5	3	5
Dieldrin	0.028	0.056	0.562	5.62	28.1	56.2	0.011	0.021	0.21	2.1	11	21

**Brominated compounds**												
BDE-47	0.009	0.018	0.178	1.78	8.9	17.8	0.005	0.009	0.09	0.9	4.5	9.0
BDE-99	0.004	0.008	0.075	0.75	3.8	7.5	0.002	0.004	0.04	0.4	2.0	4.0
BDE-100	0.002	0.004	0.038	0.38	1.9	3.8	0.001	0.002	0.02	0.2	1.0	2.0
BDE-153	0.001	0.002	0.021	0.21	1.1	2.1	0.001	0.001	0.01	0.1	0.5	1.0
BDE-154	0.002	0.003	0.03	0.30	1.5	3.0	0.001	0.002	0.02	0.2	1.0	2.0
BDE-209	0.005	0.009	0.094	0.94	4.7	9.4	0.005	0.009	0.09	0.9	4.5	9.0
HBCD	0.027	0.055	0.545	5.45	27.3	54.5	0.018	0.035	0.35	3.5	17.5	35.0

**Perfluorinated compounds**												
PFHxS	3.905	7.809	78.092	780.92	3904.6	7809.2	1.711	3.422	34.22	342.2	1711	3422
PFOS	20.761	41.522	415.221	4152.21	20761.1	41522.1	11.174	22.348	223.48	2234.8	11,174	22,348
PFOA	2.105	4.209	42.094	420.94	2104.7	4209.4	0.872	1.743	17.43	174.3	872	1743
PFNA	0.546	1.093	10.925	109.25	546.3	1092.5	0.254	0.507	5.07	50.7	254	507
PFDA	0.188	0.375	3.754	37.54	187.7	375.4	0.097	0.193	1.93	19.3	97	193
PFUnDA	0.168	0.337	3.368	33.68	168.4	336.8	0.095	0.190	1.9	19	95	190

### 2.2 Human induced pluripotent stem cell (hiPSC)-derived neural stem cells (NSCs) differentiated into mixed culture of neurons and astrocytes

IMR90 fibroblasts (Coriell, [53]) were reprogrammed into hiPSCs at I-Stem (France) by the viral transduction of Oct4 and Sox2 using pMIG vectors (Addgene). IMR90-hiPSCs were kindly provided by Prof Marc Peschanski (I-Stem, France) and were used to derive NSCs, as detailed in [54]. In brief, undifferentiated IMR90-hiPSC colonies were cut into fragments of about 200 μm x 200 μm using a 1 ml syringe with a 30 G needle, and plated in a 60 mm ultra-low attachment Petri dish (5 mL/60-mm Petri dish) to enable embryoid bodies (EBs) formation. After 2 days, floating EBs were collected and plated onto matrigel-coated 60 mm dishes (∼ 50 EBs/dish) and cultured in the presence of complete neuroepithelial induction medium [54], changing medium every other day until day 7 to allow formation of neuroepithelial aggregates (rosettes). On Day 8, rosette-like structures were isolated using a 1 ml syringe with a 30 G needle, collected by centrifugation and partially dissociated in 1 ml of 1x DPBS (without calcium and magnesium) using a 1000-μL pipette. Rosette fragments were further cultured in the presence of neural induction (NI) medium [i.e., DMEM/F12 with Glutamax (ThermoFisher), Non-Essential Amino Acids, Penicillin/Streptomycin (50 U/mL), N2 supplements, B27 supplements (without retinoic acid) (all from ThermoFisher Scientific), heparin Grade I-A (2 μg/mL, Merck), bFGF (10 ng/mL), EGF (10 ng/mL) and BDNF (2.5 ng/mL) (all three from ThermoFisher Scientific)], refreshing medium every other day. Obtained NSCs were maintained in proliferation and passaged at least 4–5 times before being differentiated into a mixed culture of neurons and astrocytes [54]. NSCs derived from neuroectodermal derivatives were cryopreserved by creating a master and working cell bank, enabling a high level of reproducibility.

To induce differentiation into a mixed culture of neurons and astrocytes, NSCs were passaged with trypsin, plated onto polystyrene Poly-d-Lysine coated 96-well (flat bottom) plates (ThermoFisher Scientific [55]) coated with reduced growth factor matrigel at a density of 7000 cells/well (i.e., 21.000 cells/cm^2^, 150 μl medium/well in 96 well plate), and differentiated for 28 days *in vitro* (DIV) in the presence of neuronal differentiation (ND) medium [i.e., Neurobasal Medium, N2 Supplements, B-27 Supplements, Penicillin/Streptomycin (50 U/mL), BDNF (2.5 ng/mL) and GDNF (1 ng/mL) (all from ThermoFisher Scientific)]. The differentiated neuronal cells are characterized by about 35–42 % glutamatergic neurons, 15–20 % GABAergic neurons, and 13–20 % dopaminergic neurons, along with 18–24 % astrocytes as previously described [54,56] and reported in this study. NSCs were exposed to POP mixtures at final concentrations 0.5x, 1x, 10x, 100x, 500x and 1000x (exposure concentrations, for analysis of viability), and 0.5x, 1x, and 1000x (for analysis of selected DNT endpoints), starting from 1 DIV for either 3, 14 or 28 days (150 μl medium/well in 96 well plate). In detail, starting from the 1000,000x ‘mother stocks’ (i.e., mixtures at 1,000,000 times human blood levels), sub-stocks solutions in DMSO were prepared (i.e., sub-stock concentrations at 500, 1000, 10,000, 100,000, and 500,000 times human blood levels) in order to have an equal final amount of solvent across all treatment conditions (i.e., 0.1 % DMSO), considering minimum of 6 technical replicates for each condition (each passage). Total medium change and treatment refreshment was performed twice a week.

### 2.3 Analysis of cell viability based on mitochondrial dehydrogenase activity using CellTiter-Blue®

After either 3, 14 or 28 days of treatment, cells were incubated with CellTiter-Blue® Reagent (1:6 dilution) for 3−4 hours in the incubator (37 °C, 5% CO_2_). After the incubation, 100 μl medium/reagent were transferred into new reading plates, and fluorescence was measured at 530–560 nm-/590 nm (excitation/emission) in a multiwell fluorimetric reader (Tecan). Results were normalized to the mean of solvent treated cells (0.1 % DMSO).

### 2.4 Immunocytochemistry (IC) and high content imaging (HCI)

**Fig. 1:**
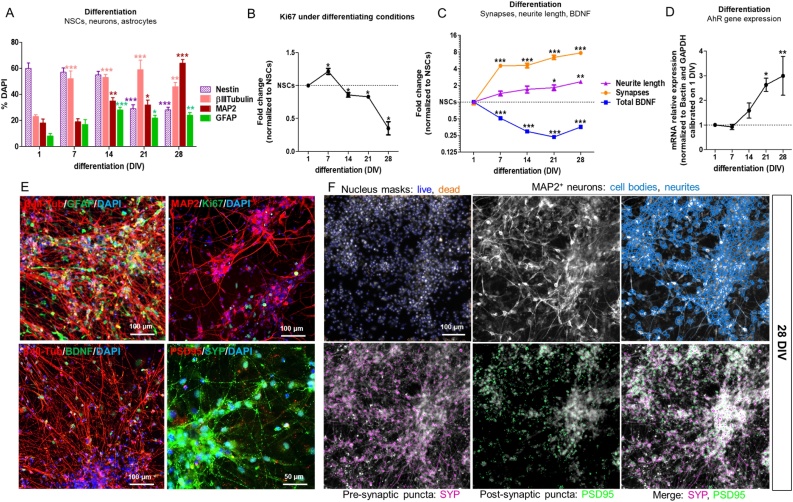
Characterization of hiPSC-derived neural stem cells (NSCs) undergoing differentiation into neurons and astrocytes in control culture. (A) Quantification of nestin, β-III-tubulin, MAP2 and GFAP positive cells shown as percentage of DAPI stained cells, comparing cells at 7, 14, 21 and 28 DIV of differentiation, to NSCs (1 DIV). (B) Quantification of Ki67+ proliferating cells undergoing differentiation as described in A. (C) Quantification of total BDNF protein levels, neurite length (β-III-tubulin staining) and the number of synapses (i.e., number of overlapping SYP/PSD95 spots in MAP2+ cells) (data in C graph were taken from [62] and presented here in a different format). (D) Gene expression analysis of AhR gene in NSCs undergoing differentiation; AhR gene expression was normalized to Bactin and GAPDH and calibrated on NSCs at 1 DIV (mean ± S.E.M. of 3 biological replicates). (E) Representative immunocytochemical images of neuronal cells and astrocytes after 28 DIV; cells were stained for β-III-tubulin (red), GFAP (green), MAP2 (red), Ki67 (green), BDNF (green), PSD95 (red) and SYP (green). (F) Representative pictures of cells at 28 DIV, stained with MAP2, synaptophysin (SYP) and PSD95 antibodies, along with DAPI to identify nuclei and identified by masks using the Thermo Scientific HCS Studio Software (Cellomics platform). Values in A, B and C are normalised to undifferentiated NSCs (1 DIV), and are shown as mean ± S.E.M. of at least 4 biological replicates (* p < 0.05, ** p < 0.01, *** p < 0.001). (For interpretation of the references to colour in the Figure, the reader is referred to the web version of this article).

After 3, 14 or 28 days of treatment with POPs (0.5x, 1x and 1000x), cells were fixed with 4% formaldehyde for 10 min and washed three times in PBS 1 × . After 15 min permeabilization with PBS 1X containing 0.1 % Triton-X-100 and 3.5 % bovine serum albumin (BSA), cells were incubated with 3.5 % BSA and 1X PBS (blocking solution) for 15 min, to prevent nonspecific binding of antibodies, and then incubated at 4 °C overnight with primary antibodies in blocking solution. For synaptogenesis analysis, cells were stained with microtubule-associated protein-2 (MAP2, chicken, 1:3000, Abcam), synaptophysin (pre-synaptic marker) (SYP, rabbit, 1:300, Abcam), and post-synaptic density protein 95 (PSD95, mouse, 1:300, Abcam) specific antibodies. Neurite outgrowth and BDNF protein levels were assessed by staining the cells with an antibody specific for β-III-tubulin (mouse, 1:500, Thermofisher), and one for BDNF (rabbit, 1:70, Thermofisher). Cells were also stained for glial fibrillary acidic protein (GFAP, chicken, 1:500, Abcam), the neural stem cell marker nestin (mouse, 1:200, Thermofisher) and the cell cycle marker Ki67 (rabbit, 1:1000, Abcam). The following day, cells were washed three times with PBS 1X and incubated for 1 h at room temperature in darkness with DyLight-conjugated secondary antibodies (1:500, Thermofisher) and nuclei counterstained with 1 μg/ml DAPI (Thermofisher). Secondary antibody incubation alone was used to determine the intensity level of fluorescent background. Mean fluorescence intensity and the relative percentages of immunocytochemically-defined cell types were quantified using the ArrayScan™ XTI High Content Platform (Cellomics) and the ArrayScan 'Neuronal Profiling V4.1′ BioApplication. This algorithm applies a specific nucleus mask around DAPI stained nuclei, distinguishing between live and pyknotic/dead cells, and a cell body mask around the cell type antibody/antigen staining (i.e., MAP2, β-III-tubulin, GFAP or Nestin). Neurite Outgrowth V.4.1 BioApplication enables the measurement of the number of neurites per neuron, the length of the neurites (expressed in μm) and neurite branching (i.e., the number of branch points per neurite) as described in [57] and [58]. The same algorithm was also used to determine synaptogenesis on the basis of the co-localised expression of SYP and PSD95 puncta, following a Thermo-Fisher standardised protocol [59] (Fig. 1F), and to quantify BDNF fluorescence intensity levels. Masks identifying live cell (non-pyknotic) nuclei, cell bodies, neurites, and other specific antigens (i.e., synapse-related proteins, BDNF, Ki67) were automatically defined using the Thermo Scientific HCS Studio Software; before automated scanning, a minimum of 7 different pictures (i.e., fields) were taken to optimize parameters for mask definition and localization. The proportion of cells positive for nestin, β-III-tub, MAP2, GFAP or Ki67 was crosschecked by manual counting of minimum 3–5 pictures to verify accuracy of automated quantification. The Cellomics platform was set up to take a minimum of 12–16 pictures/well at 10x magnification (20x and 40x magnification images were taken to show high magnification images). A minimum of 6 technical replicates for each condition were performed for each passage.

### 2.5 RNA extraction and quantitative real time PCR (qPCR)

Analysis of AhR gene expression by qPCR was performed in hiPSC-derived NSCs undergoing differentiation (at 1, 7, 14, 21 and 28 DIV), and upon treatment with *PerF + Br + Cl* at 0.5, 1 and 1000x for 3d (acute treatment), and upon treatment with all 7 tested POP mixtures at 1x concentration for 14 and 28 days vs untreated (solvent control) cells. RNA was isolated using the RNAqueous®-Micro Kit (ThermoFisher) according to manufacturer's instructions, and 500 ng of total RNA was reverse transcribed using the High Capacity cDNA Reverse Transcription Kit (as directed, ThermoFisher). qPCR reactions were run in duplicate using TaqMan® Gene Expression Master Mix (ThermoFisher) and the following TaqMan gene expression assays (all from ThermoFisher): AHR (Hs00169233_m1), ACTB (Hs99999903_m1) and GAPDH (Hs02758991_g1). Fluorescent emission was recorded in real-time using the ABI PRISM Sequence Detection System 7900 H T (ThermoFisher). PCR amplification conditions consisted of 45 cycles with primers annealing at 60 °C. Relative RNA quantities were normalized to the reference genes GAPDH and Bactin and NSCs (1 DIV) or solvent control cells (0.1 % DMSO, at 14 and 28 days) were used as calibrating conditions (ΔΔCt method).

### 2.6 Mathematical modelling to predict mixture combined effects

To investigate whether complex POP mixtures induced additive, synergistic, or antagonistic effects on the selected DNT endpoints, a mathematical modelling focussing on the pairwise combination of the different classes of tested POPs (i.e., *PerF + Br*, *PerF + Cl*, and *Br + Cl*) was applied. For each endpoint, the dose response of complex mixture containing solely *PerF*, *Br*, or *Cl* compounds was modelled using a nonlinear least square estimation for each DNT endpoint of interest. In a second step, the theoretical non-interaction surface of response was calculated by applying the Loewe additive model for each pair of chemical classes by using the R packages BIGL [60] and DrugCombo [61]. The Loewe additivity equation provides a prediction of the dose combination eliciting a given additive effect. For each dose combination, the Z-score (i.e., standardized difference between the observed effect and the effect predicted by a generalized Loewe model) was calculated, and the mean of multiple standardized differences was evaluated to informally compare the predicted and observed values. Departure from the null model may indicate potentiated (synergistic or antagonistic) effects.

### 2.7 Statistical analysis

Statistical significance was assessed by one-way ANOVA with Dunnett's Multiple Comparison Test as Post Test (for all graphs, unless otherwise indicated in figure legend), or one-tailed paired *t*-test (for graph in Supplementary Fig. 1B) comparing all conditions vs solvent control (Ctr, 0.1 % DMSO) or vs NSCs (undifferentiated cells) using JMP®Pro, Version 14 (SAS Institute Inc., Cary, NC), or GraphPad Prism 5 (https://www.graphpad.com/). All data represent the average of 4 biological replicates (i.e., average of 3−4 passages, with a minimum of 6 technical replicates (wells) each passage ± standard error mean (S.E.M.). For all graphs, an asterisk over a data point indicates a significant difference with the solvent control group (* p < 0.05, ** p < 0.01, *** p < 0.001).

## 3 Results

### 3.1 Human iPSC-derived NSCs undergoing differentiation into mixed culture of neurons and astrocytes: characterisation of synaptogenesis, neurite outgrowth, BDNF protein levels, and Aryl hydrocarbon Receptor (AhR) gene expression

Analysis of synaptogenesis-related proteins, synaptophysin (SYP, pre-synaptic) and post-synaptic density protein 95 (PSD95), along with the dendritic marker microtubule-associated protein-2 (MAP2), neurite outgrowth (assessed via β-III-tubulin staining), and BDNF protein levels was performed using hiPSC-derived NSCs undergoing differentiation for 28 days, as previously described [62]. Both neuronal markers, β-III-tubulin and MAP2 increased over the time course of neuronal differentiation (approximately 46 % for β-III-tubulin + cells, and 64 % for MAP2+ cells at 28 DIV), with a progressive increase of neurite length of about 2.3 fold compared to undifferentiated cells (Fig. 1A, C, E). As expected, the cell cycle marker Ki67 (indicative of proliferation) decreased upon differentiation (∼65 % decrease at 28 DIV compared to undifferentiated cells) (Fig. 1B, E). After 28 DIV, the presence of about 24 % astrocytes was observed (i.e., cells expressing the astrocytic marker glial fibrillary acidic protein, GFAP) as well as ∼29 % of nestin+ cells, indicative of NSCs (Fig. 1A, E, and Supplementary Table 2). Moreover, as described in our previous study [62] and reported in Fig. 1C for ease of reading, SYP and PSD95 co-localization, biomarkers of synapse formation, increased by ∼7.7 fold after 28 DIV of differentiation (Fig. 1C, E, F), whilst BDNF protein levels decreased over time (∼65 % reduction compared to undifferentiated cells), being more expressed in proliferating NSCs (Fig. 1C, E).

As AhR has been shown to regulate neuronal differentiation in different models [41], we characterized the expression of AhR gene in NSCs undergoing differentiation. Upregulation of AhR gene expression occurred starting from 14 DIV, becoming statistically significant after 21 DIV (∼2.5-fold increase compared to NSCs at 1 DIV) (Fig. 1D).

### 3.2 Effects of an acute treatment (3 days) with PerF + Br + Cl POP mixture

#### 3.2.1 Cell Viability, proliferation and proportions of diverse cell types

**Fig. 2:**
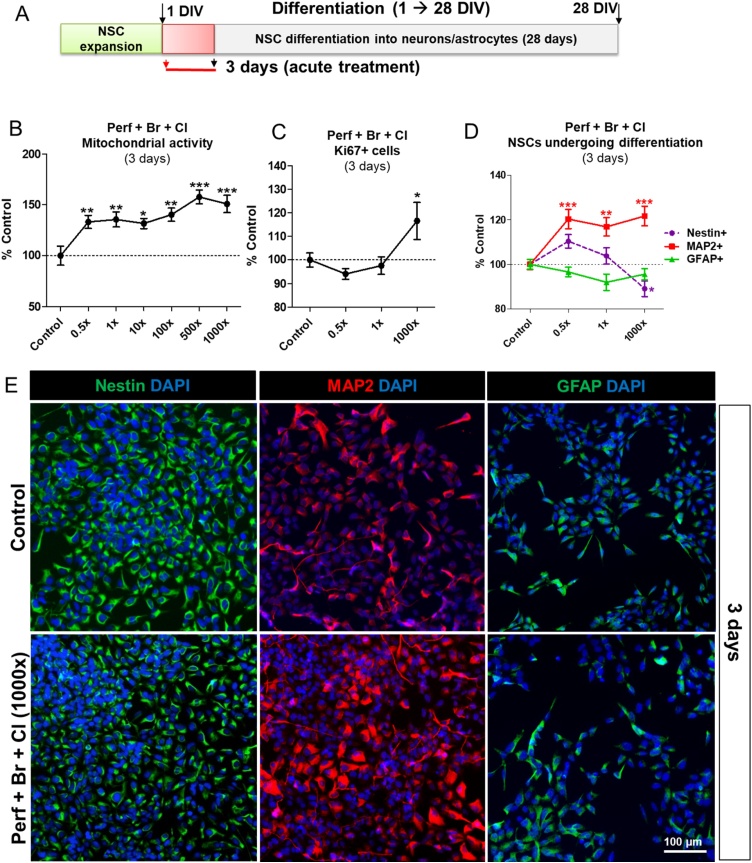
Effects of an acute treatment (3 days) with POP mixture containing PerF + Br + Cl on cell viability, proliferation and proportions of cell populations. (A) Starting on differentiation day 1 (1 DIV), hiPSC-derived NSCs were treated for 3 days with *PerF + Br + Cl* mixture at the concentrations 0.5, 1, 10, 100, 500 and 1000x (see Table 1), and analysis of mitochondrial activity by means of resazurin test was performed (B). (C) Quantification of Ki67+ proliferating cells, and (D) nestin, MAP2 and GFAP positive cell percentages upon treatment with *PerF + Br + Cl* mixture at the concentrations 0.5, 1, and 1000 × . (E) Representative immunocytochemical images of NSCs (nestin+, green), and NSCs undergoing differentiation toward neurons (MAP2+, red) and astrocytes (GFAP+, green) in solvent control culture (upper panels) and upon 3 day treatment with *PerF + Br + Cl* mixture at 1000x concentration (10x magnification images). Values in B, C and D are normalised to solvent control cells and are shown as mean ± S.E.M. of 4 biological replicates (* p < 0.05, ** p < 0.01, *** p < 0.001). (For interpretation of the references to colour in the Figure, the reader is referred to the web version of this article).

We initially assessed the effects of an acute exposure (3 DIV) to the POP mixture containing all 29 perfluorinated (*PerF*), brominated (*Br*), and chlorinated (*Cl*) compounds (*PerF + Br + Cl* mixture) (Fig. 2A), to assess whether the most complex mixture was cytotoxic to NSCs at an early stage of differentiation. After 1 DIV, cells were exposed to 0.5x, 1x, 10x, 100x, 500x and 1000x the concentrations of POPs found in human blood (Table 1). After 3 days, no cytotoxic effects were observed and, on the contrary, an increase of mitochondrial activity was detected with all tested concentrations (Fig. 2B).

We further analysed the effects of *PerF + Br + Cl* mixture at three concentrations (0.5x, 1x and 1000x) on Ki67, a marker of cell proliferation, expressed by cells in G1, S, G2 and M phase, and we observed about 10–15 % increase of Ki67+ cell percentage upon exposure only to the highest tested concentration (1000x) (Fig. 2C).

However, a higher percentage of NSCs undergoing neuronal differentiation (positive for MAP2) was found with all tested concentrations (∼ 20 % increase compared to solvent control treated cells). Additionally, a modest decrease of nestin + NSC percentage was recorded upon treatment with 1000x (∼ 10 % decrease compared to control), whilst the percentage of cells undergoing differentiation toward astrocytes (positive for GFAP) did not significantly change (Fig. 2D, E).

#### 3.2.2 Synapse-related markers, neurite outgrowth, BDNF protein levels and AhR gene expression

**Fig. 3:**
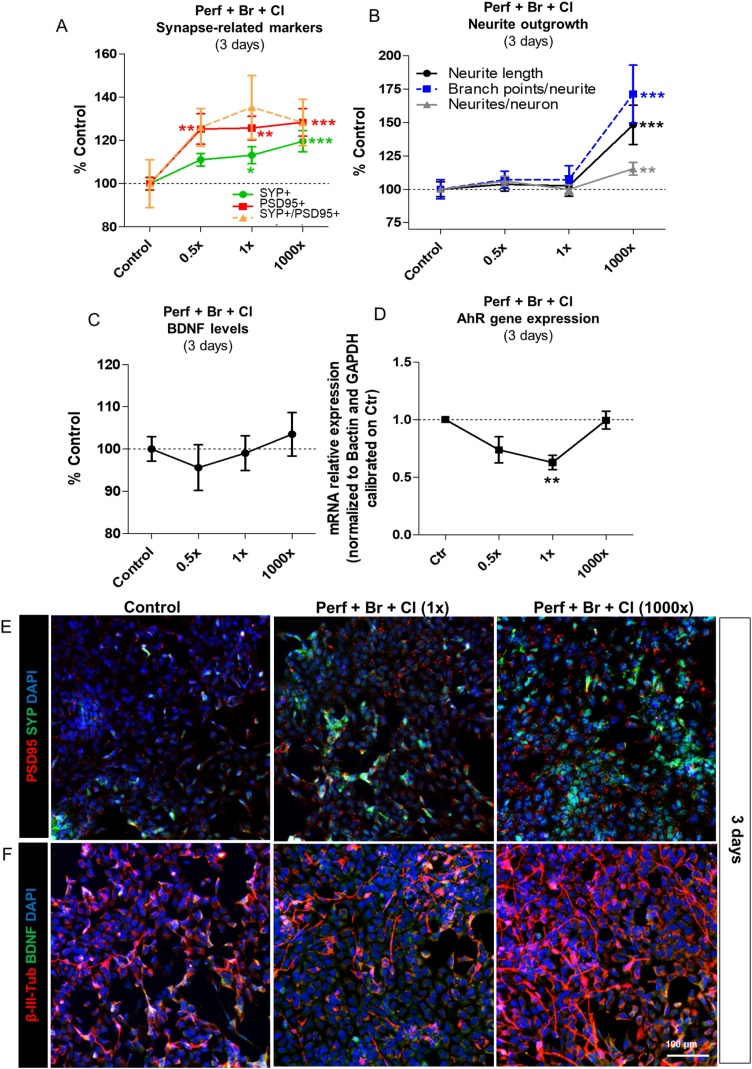
Effects of an acute treatment (3 days) with POP mixture containing PerF + Br + Cl on synapse-related markers, neurite outgrowth, BDNF protein levels and AhR gene expression. (A) Quantification of synapse-related markers SYP (green) and PSD95 (red) staining, and the number of overlapping SYP+/PSD95+ spots (orange), (B) neurite outgrowth (neurite length (black), number of branch points/neurite (dashed blue), and number of neurites/neuron (grey)), and (C) total BDNF protein levels, upon 3 day treatment with *PerF + Br + Cl* mixture at 0.5, 1, and 1000x concentrations. (D) Gene expression analysis of AhR gene upon 3 day-treatment with with *PerF + Br + Cl* mixture at 0.5, 1, and 1000x concentrations; AhR gene expression was normalized to Bactin and GAPDH and calibrated on solvent control cells (mean ± S.E.M. of 3 biological replicates). (E, F) Representative immunocytochemical images of control culture, and cells treated with *PerF + Br + Cl* mixture at 1x and 1000x concentrations. Cells were stained for PSD95 (red) and SYP (green) (E), neurite outgrowth (β-III-tubulin, red), and BDNF (green) (F). Values in A, B and C are normalised to solvent control cells and are shown as mean ± S.E.M. of 4 biological replicates (* p < 0.05, ** p < 0.01, *** p < 0.001). (For interpretation of the references to colour in the Figure, the reader is referred to the web version of this article).

The effects induced by a 3-day treatment with *PerF + Br + Cl* mixture on the level of synapse-related markers, neurite outgrowth and BDNF protein levels were also assessed. About a 20–25 % increase of both SYP (pre-synaptic) and PSD95 (post-synaptic) protein levels, along with a trend towards an increase of SYP+/PSD95+ overlapping spots (not significant) was visible after 3-day treatment with all tested POP mixture concentrations (0.5x, 1x and 1000x) (Fig. 3A, E). An increase of all neurite features (neurite length, number of branch points per neurite, and the number of neurites per neuron) was observed upon exposure to the highest concentration (1000x) (Fig. 3B, F), while BDNF protein levels did not significantly change under these conditions (Fig. 3C, F).

Analysis of AhR gene expression revealed a decrease upon treatment with *PerF + Br + Cl* mixture at lower concentrations (p < 0.01, 1x concentration), while the highest tested concentration (1000x) did not show differences compared to solvent control cells (Fig. 3D).

### 3.3 Effects of a repeated dose exposure (14 days or 28 days) to different POP mixtures

**Fig. 4:**
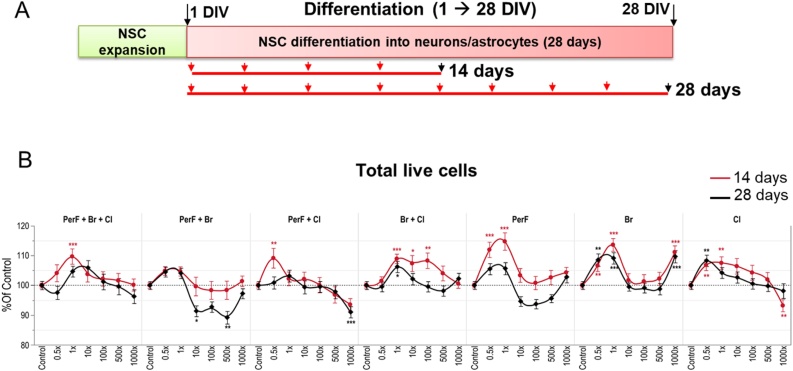
Effects of a repeated dose exposure (14d and 28d) to different types of POP mixtures on cell viability. (A) Starting on differentiation day 1 (1 DIV), hiPSC-derived NSCs were treated for either 14 or 28 days with a mixture containing *PerF + Br + Cl* compounds, and sub-mixtures containing *Cl* only, *Br* only, *PerF* only, *Br + Cl*, *PerF + Br*, and *PerF + Cl* compounds, at the concentrations 0.5, 1, 10, 100, 500 and 1000x (Table 1). (B) Total number of live (non-pyknotic) cells was quantified by DAPI staining. Data are normalized to solvent control (0.1 % DMSO) and presented as mean ± S.E.M., with asterisks indicating statistical significance compared to solvent control (* p < 0.05, ** p < 0.01, *** p < 0.001).

We further assessed the effects induced by different POP mixture compositions to investigate contributions of individual POP chemical groups to DNT effects. To study this, six additional mixtures were tested (Table 1), and cells were treated starting from 1 DIV, for either 14 or 28 days (Fig. 4A).

#### 3.3.1 Cell viability assay

In these experiments, based on CellTiter blue viability assay, no cytotoxic effects were observed (data not shown). On the other hand, an increase of total live cells (assessed by automated quantification of cells with non-pyknotic (live) nuclei by DAPI staining) of about 10 % after 14-day treatment was observed in cells treated with *PerF + Br + Cl, PerF + Cl, Br + Cl, PerF* only, *Br only* and *Cl only* at 0.5x and 1x human concentrations (Fig. 4B, red curves). After 28 days, these effects were in some cases mitigated (e.g., *PerF only*), a modest decrease of live cells (about 9–12 %) was observed upon treatment with *PerF + Br* (at 10x and 500x), while a slightly higher number of live cells (about 8–10 %) was found upon exposure to *Br + Cl*, or *Br only* and *Cl only* at low concentrations (0.5x and 1x) (Fig. 4B, black curves).

#### 3.3.2 Analysis of different cell type populations (NSCs, neurons and astrocytes)

Considering that most of POP mixture effects on live cell number were observed upon treatments with lower concentrations, we performed subsequent analyses of POP mixture effects using the two lowest concentrations (0.5x and 1x) and the highest one (1000x) for comparative purposes.

**Fig. 5:**
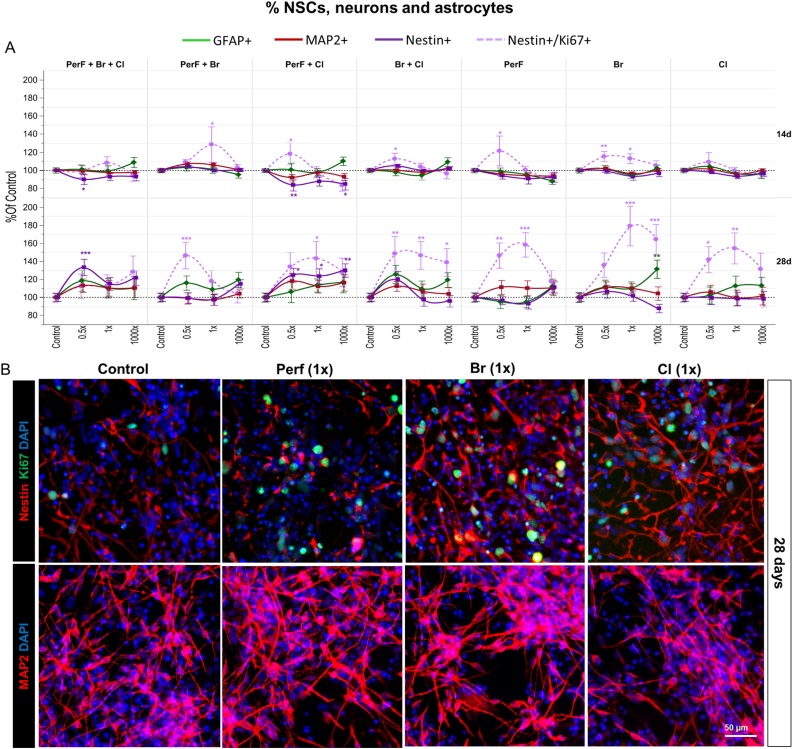
Effects of a repeated dose exposure (14d and 28d) to different types of POP mixtures on proportion of diverse cell populations. (A) Quantification of nestin+, nestin+/Ki67+, MAP2+ and GFAP + cell percentages upon 14 or 28 day treatment with POP mixtures at the concentrations 0.5, 1, and 1000 × . (B) Representative immunocytochemical images of nestin (red) and Ki67 (green) (upper panels), and MAP2 (red) staining (lower panels) in solvent control culture and upon 28 day treatment with PerF only, Br only and Cl only mixtures at 1x concentration. Data are normalized to solvent control (0.1 % DMSO) and presented as mean ± S.E.M. with asterisks indicating statistical significance compared to solvent control (* p < 0.05, ** p < 0.01, *** p < 0.001). (For interpretation of the references to colour in the Figure, the reader is referred to the web version of this article).

To assess the proportion of proliferating NSCs, we analysed the percentage of double positive nestin^+^/Ki67^+^ cells upon treatment with the seven POP mixtures. After 14-day treatment, the overall percentage of nestin+ cells was slightly decreased (about 15 %) only upon treatment with *PerF + Cl* mixture (Fig. 5A, violet curves). However, the percentage of nestin+/Ki67+ showed a tendency towards an increase by 10–12 % upon treatments with most tested POP mixtures (*PerF + Br*, *PerF + Cl*, *Br + Cl*, *PerF only*, and *Br only*) at 0.5x and 1x concentrations (Fig. 5A, pink dashed curves). Interestingly, after prolonged treatment (28 days), a more remarkable increase of proliferating NSC (nestin+/Ki67+) percentage (∼ 40–70% increase compared to solvent control) was found upon treatment with all tested POP mixtures (Fig. 5A and B, pink dashed curves), except for the mixture containing all 29 POPs (*PerF + Br + Cl*), which induced about 30 % increase in the overall nestin+ cell percentage (Fig. 5A, violet curves).

While the proportion of MAP2+ cells did not significantly change upon 14-day treatment with all POP mixtures, after 28 days, a modest (although not significant) increase of MAP2^+^ neuronal cell percentage was observed, except in cells treated with *PerF + Br* and *Cl* only chemicals (Fig. 5A and B, red curves).

Quantification of astrocytes by immunostaining with a GFAP antibody indicated that no significant change in cell percentage compared to solvent control occurred after 14-day treatment in any mixtures or concentrations (Fig. 5A, green curves).

After 28 days of treatment, a tendency towards a modest increase in the percentage of astrocytes was observed for all mixtures, with a statistically significant increase of approximately 30 % in astrocyte cell numbers in culture exposed to *Br only* at 1000x concentration (Fig. 5A, green curves). Altogether, these analyses suggest that, in the long term treatment (28 days), *PerF, Br*, and *Cl* compounds and their combined mixtures (i.e., *PerF + Br, PerF + Cl*, *Br + Cl*) at concentrations found in human blood or below (0.5 and 1x), cause an increase of proliferating NSCs.

#### 3.3.3 Analysis of synaptogenesis, neurite outgrowth and BDNF protein levels

We further investigated whether POP mixtures perturbed synaptogenesis, neurite length, and BDNF protein levels.

##### 3.3.3.1 Synaptogenesis

**Fig. 6:**
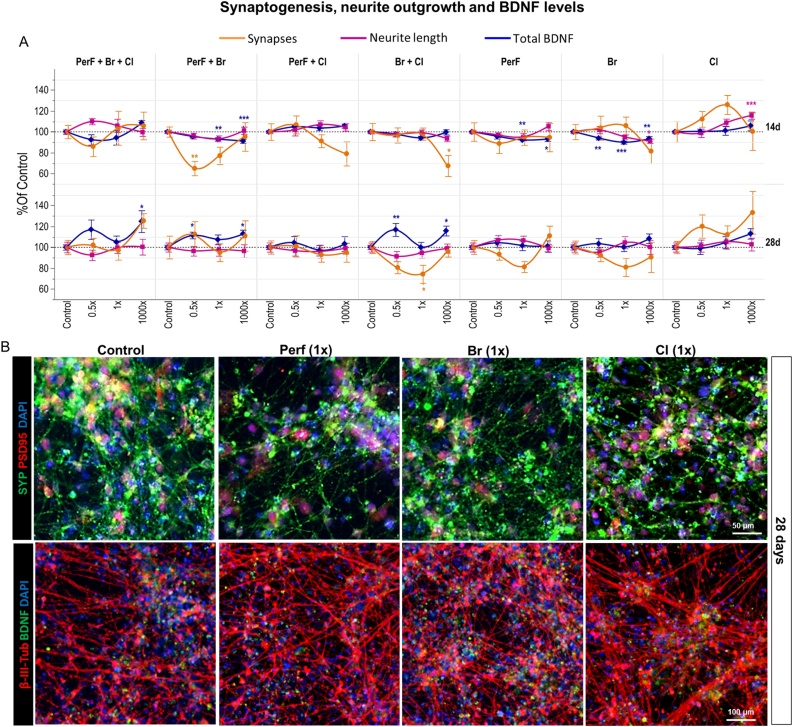
Effects of a repeated dose exposure (14d and 28d) to different types of POP mixtures on synaptogenesis, neurite outgrowth and BDNF levels. (A) Quantification of synapses (i.e., overlapping SYP/PSD95 spots) (orange), neurite length (purple) and BDNF protein levels (blue), upon 14 or 28 day treatment with POP mixtures at the concentrations 0.5, 1, and 1000 × . (B) Representative immunocytochemical images of PSD95 (red) and SYP (green) (upper panels), and β-III-tubulin (red) and BDNF (green) staining (lower panels) in solvent control culture and upon 28 day treatment with *PerF* only, *Br* only and *Cl* only mixtures at 1x concentration. Data are normalized to solvent control (0.1 % DMSO) and presented as mean ± S.E.M. with asterisks indicating statistical significance compared to solvent control (* p < 0.05, ** p < 0.01, *** p < 0.001). (For interpretation of the references to colour in the Figure, the reader is referred to the web version of this article).

After 14-day treatment, ∼ 33–38 % decrease in the number of synapses was observed upon treatment with *PerF + Br* at 0.5x concentration (Fig. 6A, orange curves). A tendency towards a decrease in synapses was also observed in cells treated with *PerF + Br + Cl* at 0.5x concentration, and upon treatment with *PerF + Cl* and *Br + Cl* mixtures at 1000x concentration (Fig. 6A, orange curves). Conversely, a tendency towards an increase of synapses was observed upon treatment with *Cl* only compounds at lower tested concentrations (not significant).

After 28 days, some of these effects were reverted to control level (e.g., *PerF + Br* and *PerF + Br + Cl*), which suggests induction of compensatory/recovery mechanisms (Fig. 6A, orange curves). While the mixture containing *PerF only* tended to decrease and the mixture with *Cl only* tended to increase the number of synapses (Fig. 6A, orange curves, and 6B), *PerF + Cl* combined together were found to have no effect on the number of synapses. On the other hand, while the *Br only* generally decreased the number of synapses and *Cl only* increased them (Fig. 6A, orange curves), their combination in the *Br + Cl* mixture was found to generally decrease synapses formation after 28 days at 0.5x and 1x human concentrations. This suggests that in presence of combined *Br + Cl*, the *Br* compounds might be the major drivers of mixture effects.

##### 3.3.3.2 Neurite outgrowth

After 14 days of exposure, ∼ 15–17 % increase of neurite length occurred upon treatment with mixture containing *Cl* compounds at 1000x concentration. On the other hand, *PerF only* and *Br only* and all other combined mixtures, showed no significant effects on neurite length (Fig. 6A, purple curves).

Prolonged treatment analysis (28 days), showed very modest, and not significant changes of neurite length upon treatment with any mixture (Fig. 6A, purple curves, and 6B).

##### 3.3.3.3 BDNF protein levels

After 14-day treatment, *PerF only* and *Br only* mixtures induced 7–9 % decrease of BDNF protein levels (Fig. 6A, blue curves). The mixture containing *PerF + Br* was found to decrease BDNF at levels comparable to *PerF only* and *Br only*, suggesting no combined effects of these chemical groups under these treatment conditions. The remaining mixtures containing chlorinated compounds (*PerF + Br + Cl, PerF + Cl*, *Br + Cl* and *Cl only*) all showed no significant changes in BDNF levels compared to the solvent control (Fig. 6A, blue curves).

Long term treatment (28 days), at 0.5x and 1x concentrations, showed no effects on BDNF under exposure to mixtures of *PerF only*, *Br only* and *Cl only* (Fig. 6A, blue curves, and 6B). When combined (*PerF + Br* and *Br + Cl* mixtures at 0.5x concentrations), a 10–15 % increase of BDNF levels was observed. Similar effect on BDNF protein levels was also observed in the total mixture (*PerF + Br + Cl*), although not statistically significant (Fig. 6A, blue curves).

Finally, we analysed AhR gene expression upon 14 and 28-day treatment with all seven POP mixtures at blood level (1x) concentration. Data indicated a tendency towards increase in AhR gene expression upon treatment with *Cl* mixture (after 14 days, red bars) and *Br* mixture (after 28 days, black bars) compared to solvent control cells at the respective time points (not significant) (Supplementary Fig. 1A). Further analysis of AhR gene expression comparing solvent control culture and cells treated with *PerF + Br + Cl* mixture at 1x concentration after 3, 14 and 28 DIV, indicated that exposure to POP mixture did not prevent the observed increase of AhR gene expression occurring during differentiation (Supplementary Fig. 1B).

**Fig. 7:**
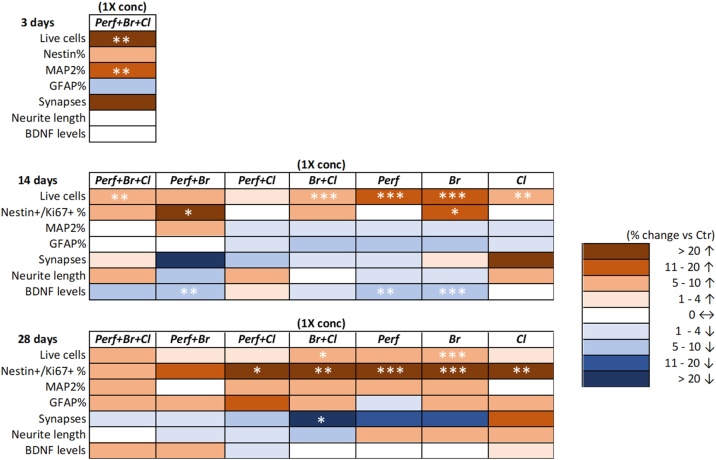
Heat map summarizing the effects of an acute (3d) and a repeated dose exposure (14d and 28d) to different types of POP mixtures at 1x concentrations (brown: increase; blue: decrease). Asterisks indicate statistical significance compared to solvent control (* p < 0.05, ** p < 0.01, *** p < 0.001). (For interpretation of the references to colour in the Figure, the reader is referred to the web version of this article).

Fig. 7 summarizes data on viability and the analyzed DNT endpoints, reporting delta values (% difference vs control) between 1x concentration *vs* control (different colors refer to ranges of delta values), obtained upon acute treatment (3 days) with the mixtures containing all 29 POPs, and repeated dose treatments (14 and 28 days) with all seven POP mixtures.

### 3.4 Mathematical modelling of POP mixture effects on selected DNT endpoints

**Fig. 8:**
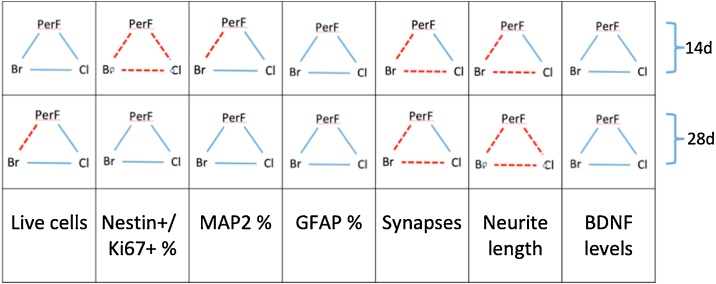
Summary of the effects observed in pairwise combinations of different classes of tested POP mixtures by mathematical modelling. Each square summarizes the effects induced by the pairwise combinations of different classes of POPs (i.e., *PerF + Br*, *PerF + Cl*, or *Br + Cl)* on the selected DNT endpoints after 14 days and 28 days of treatment with the three tested concentrations (0.5, 1 and 1000x). Blue lines indicate additive effects observed with all the three concentrations, according to the Loewe non-interaction model. Red dashed lines represent potentiated (synergistic or antagonistic) effects observed with at least one of the three tested concentrations of combined POPs mixtures. (For interpretation of the references to colour in the Figure, the reader is referred to the web version of this article).

The comparison between the experimental observations and the mathematically predicted non-interaction surface, reveals that co-treatments with different classes of POPs combined in complex mixtures, in general elicited an additive response on the selected DNT endpoints (blue lines, Fig. 8). For this analysis we took into account all three tested concentrations (0.5x, 1x and 1000x) and solvent control in order to establish, according to the Loewe non-interaction model, whether two POP classes mixed together induced effects similar to (additivity) or greater than (synergism or antagonism) the null reference model (any departure from the null model may indicate potentiated, i.e., synergistic or antagonistic, effects). Indeed, according to the 2019 EFSA guidance on mixture [63], the so-called additivity or non-interaction assumption applies to chemicals in a mixture that exert their effects without diminishing or enhancing each other’s toxicity.

**Fig. 9:**
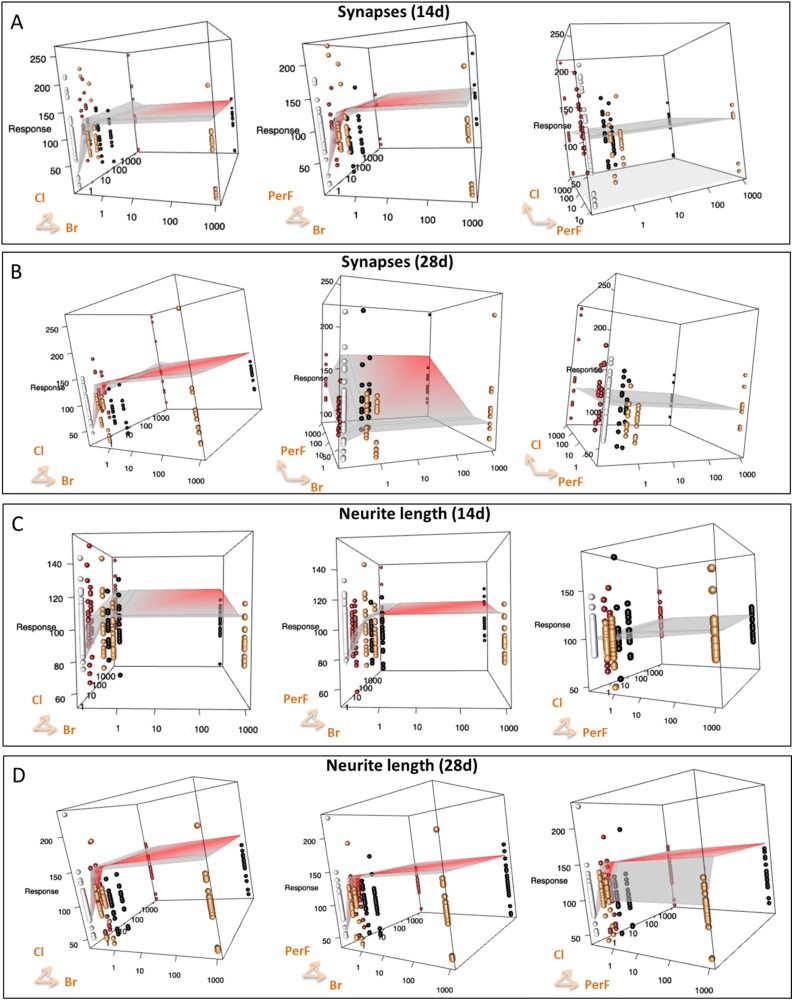
Comparison between predicted and observed effects induced by the combination of different classes of POPs on the number of synapses (A and B) and neurite length (C and D). Panels A and C refer to 14 days of treatment, Panel B and D to 28 days. In each plot, points refer to the observed effects (yellow and red dots correspond to the mixtures composed of one class of chemicals, black dots are the values observed by testing the complex mixture including the two classes of chemicals, white dots correspond to solvent control), whereas the grey surface corresponds to the model-predicted response. The surface is colored according to the median Z-scores; the red surface indicates possible synergistic or antagonistic (potentiated) effects. (For interpretation of the references to colour in the Figure, the reader is referred to the web version of this article).

When the experimental data significantly deviate from the theoretical non-interaction values predicted by the Loewe model, this indicates potentiated (synergistic or antagonistic) effects (red dashed lines, Fig. 8). In particular, after 14 days, all the tested pairwise combinations of POPs classes affected the percentage of nestin+/Ki67+ cells beyond the null model (Fig. 8). On the other hand, the combination of *PerF + Br* at the highest tested concentration (1000x) altered the percentage of MAP2+ cells in an antagonistic manner after 14 days (compared to *PerF* only and *Br* only mixtures), while after 28 days caused a modest decrease of total live cell number in a synergistic manner. Additive effects of *PerF + Br* mixture could be observed only at physiological concentrations (0.5 and 1x). Despite high variability of data, the DNT endpoints that resulted more sensitive to the combined effects of different classes of POPs appeared to be synaptogenesis (number of synapses) and neurite outgrowth (neurite length). Both endpoints were perturbed in a potentiated manner in particular by the combination of *PerF + Br* and *Br + Cl* mixtures after both time points (14 and 28 days) (Fig. 8, red dashed lines, and Fig. 9, red surfaces).

## 4 Discussion

Epidemiological studies have indicated that diverse POP mixtures may influence brain development since exposed children exhibit neuropsychological deficits, including lower IQ, impaired learning, memory and attention, in addition to motor deficits [[28], [29], [30], [31],^64^]. However, neurodevelopmental processes and mechanisms involved and their possible association with neurodevelopmental disorders are still not fully understood. Our study provides some new insights about the biological plausibility between exposure to POPs at human relevant concentrations and disturbance of neurodevelopmental processes, central for normal brain development. Based on the obtained results, POP mixtures corresponding to typical concentrations found in the blood of a Scandinavian population [37], show tendency towards impairment of some key neurodevelopmental processes, such as cell proliferation, neuronal differentiation and synaptogenesis at concentrations as low as 0.5x and 1x human blood levels. In line with the observed effects at very low concentrations, the same POP mixtures (*Cl* and *PerF*) at 0.5x and 1x human blood levels were recently found to induce respiratory burst in human monocytes and lymphocytes *in vitro* [65]. The DNT effects in the current study entailed alterations in neuronal cell proportions, neurite outgrowth, expression of biomarkers of synapse formation and BDNF protein levels using mixed cultures of human neuronal and glial cells obtained from hiPSC-derived NSCs. Such cell models are considered suitable for DNT testing [[66], [67], [68]], as under *in vitro* conditions they can mimic key neurodevelopmental processes, critical for normal human brain development [69,70].

### 4.1 POP mixtures effects on synaptogenesis and neurite outgrowth

At an early stage of differentiation (1 DIV), NSCs exposed for 3 days to the mixture containing all 29 compounds (*PerF + Br + Cl)* showed an increase (about 10–25 %) of synapse-related markers (SYP, PSD95 and their co-localization) with all tested concentrations (0.5x, 1x and 1000x), which suggests a possible increase of synaptogenesis.

It should be noticed that individual concentrations of congeners tested in this study, especially at 0.5x and 1x concentrations (Table 1), are relatively and often lower than those tested in other *in vitro* studies, e.g., 24-h treatment with 10–250 μM PFOS and PFOAS on PC12 cells [71]; 11-day treatment with 10–1000 nM BDE-209 and 0.01−5 μM BDE-47 on human ESCs undergoing neuroectodermal differentiation [72]; long-term exposure to 300 nM PCB-52 and 2 nM PCB-138 or -180 in rat cerebellar neurons [73]. According to a 2016 study, in Chinese occupationally exposed workers, levels of detected PFOS in serum were up to 118,000 ng/ml (236 μM) [74], suggesting that exposure to such very high levels (higher than those tested in the present study) may actually occur. Furthermore, measurements of organochlorine (OC) or PBDE concentrations in human brain tissues [75] associated with neurological disorders [[76], [77], [78], [79]] have been performed. OC concentrations tested in our recent experiments [50] reflected OC levels found in human brains. Such levels were comparable with those in the lowest dose exposed offspring (exhibiting disturbed hippocampal gene expression), confirming human relevance of the exposure.

By extending the exposure time (14 days), the observed effects almost reverted back to control levels, and a tendency towards an increase of synapse number (about 20 % at 1x concentration) was only observed upon treatment with *Cl only* mixture, while other POP mixtures generally caused a decrease of synapses observed at low concentrations (*Perf + Br*, at 0.5x) or higher concentrations *(Perf + Cl, Br + Cl* at 1000x) (Fig. 6A, 14d).

The increase of synapses observed upon treatment with *Cl only* mixtures at 1x concentration after 14 days of exposure, correlated with the tendency towards increased neurite length (with *Cl only* mixture at 1000x), which serves as post-synaptic compartment for synapse formation (Figs. 6A and 7, 14d).

After a prolonged exposure (28 days), a decrease of synapse number was observed upon treatment with *PerF only, Br only*, and *Br + Cl* at 1x concentration (Figs. 6A and 7, 28d), except for the mixture containing *Cl only* compounds, which induced a modest (not significant) upregulation (> 20–30 %) of synapse number at all tested concentrations (Figs. 6A and 7, 28d). These data suggest that *Cl only* compounds could be the main triggers of synapse increase in long term treatments, while exposure to *Perf* and *Br* compounds tends to decrease synaptogenesis after prolonged exposure time.

Dysregulation of synaptogenesis (both increase and decrease) upon exposure to *Cl* POPs has also been reported in *in vivo* studies; for instance, downregulation of both SYP and PSD95 has been shown upon perinatal exposure to PCB mixtures (PCB 28, 52, 101, 138, 153, and 180) [80], while increase of synaptogenesis has been reported upon perinatal exposure to PCB 95 [81]. One of the possible mechanisms by which developmental exposure to PCBs (PCB 52, 138, 180) may mediate impairment of cognitive function in rats or humans, could be through reduction of glutamate-NO-cGMP signaling [73].

With regard to *PerF* compounds, opposite to our long term exposure studies, increase of synaptogenesis has been observed in neonatal mice treated with a single oral dose of *PerF* compounds PFOS and PFOA, where increased levels of synaptogenesis-related proteins (CaMKII, Tau, GAP-43, and SYP) were found in the hippocampus and cerebral cortex, and were linked to behavioral alterations and changes in the cholinergic system [82,83].

Prenatal exposure to PFOS (from gestational day 1 to postnatal day (PND) 7) was found to decrease the expression of synapse-related proteins (NGFR, TrkC, and VGLUT2) in neonatal rats, and these effects were associated with down-regulation of miRNAs (miR-466b, −672, and −297) involved in synapse transmission [84]. Similarly, offspring of rat dams PFOS-treated during gestation (0.1, 0.6, and 2.0 mg/kg birth weight, from gestation day 0 to 20) showed decreased mRNA levels of synapsin -1 and -2, and SYP [85]. Moreover, prolonged treatment with PFOS was found to strongly inhibit synaptogenesis in cultured hippocampal neurons through enhancement of Ca^2+^ channels function [86].

Concerning *Br* compounds, there are limited and conflicting reports regarding their effects on synaptogenesis. For instance, BDE-209 was found to upregulate the amount of SYP in the hippocampus (but not in the cerebral cortex) of neonatal mice exposed for 7 days to BDE-209 [87], and to downregulate SYP expression in the same brain region of neonatal rats exposed for 5 days to BDE-209 [88].

BDE-47, the most prevalent brominated congener, and its hydroxylated metabolite (6OH-BDE-47), were reported to disrupt synapse development and neuronal cell maturation in embryonic rat cortical neurons, via alteration of chromatin remodeling and gene expression [89]. These data support our *in vitro* studies, as BDEs also decreased the number of synapses (*Br + Cl* and *Br* only at 1x concentration) especially after 28 days of exposure.

With regard to neurite outgrowth, prolonged exposure to POP mixtures did not cause significant alterations of neurite length compared to untreated cells with all tested conditions, except for mixture with *Cl only* compounds after 14d, which was found to significantly increase (by about 12 %) the length of neurites but only at the highest tested concentration (1000x) (Figs. 6A and 7, 14d), which is anyway relatively low or comparable to concentrations of individual congeners tested in other *in vitro* studies (e.g., [[71], [72], [73]].

Similarly, Aroclor 1254 (a mixture of PCB congeners) has been shown to increase dendritic growth in CA1 hippocampal pyramidal neurons and cerebellar Purkinje cells in perinatally exposed male rats at PND 60 [90], and to increase basal dendritic arborization, but inhibit experience-dependent dendritic growth in cerebellar Purkinje cells and neocortical pyramidal neurons in perinatally exposed male rats at PND 31 [91].

While our data are inconclusive with respect to *PerF* or *Br* effects on neurite outgrowth, other *in vitro* studies have reported decrease of neurite outgrowth occurring upon exposure to *PerF* compounds. For instance, a reduction of neurite length has been shown upon prolonged treatment with PFOA in cultured rat hippocampal neurons [92]. Along the same line, decrease of neurite outgrowth has been shown upon treatment with PFOS in rat primary cortical neurons, and when co-cultured with astrocytes, these effects were alleviated [93], supporting the important role of astrocytes in neuronal differentiation.

Additionally, BDEs and their hydroxylated metabolites have been shown to bind to thyroid hormone receptors and transport proteins, disrupt Ca² homeostasis, and modulate GABA and nicotinic acetylcholine receptor signaling [94]. BDE-99 [95] and BDE-209 [96] have been reported to shorten neurite length in PC12 cells and NSCs undergoing differentiation *in vitro*.

According to the Loewe non-interaction model, co-treatments with different classes of POPs combined in complex mixtures generally elicited an additive response on the selected DNT endpoints (blue lines, Fig. 8). Mathematical modelling suggests that both synaptogenesis (number of synapses based on co-localization of pre and postsynaptic protein) and neurite outgrowth (neurite length) were the most sensitive DNT endpoints, with the *PerF + Br* and *Br + Cl* mixtures inducing potentiated effects after 14 and 28-day treatment (Fig. 9). The high variability of data observed also in control culture (white dots in Fig. 9) may be due to the dynamic processes taking place during neuronal differentiation.

In line with our previous study [62], the DNT effects induced by e.g. *Cl only*, possibly characterized by similar mode of action (MoA), can be greater than the effects induced by more complex mixtures, accounting for a higher number of different chemicals covering different MoA (e.g., *PerF + Br + Cl*).

### 4.2 POPs and dysregulation of brain derived neurotrophic factor

One of the key neurotrophic factors for brain development is BDNF, which promotes neuronal survival, differentiation, including neurite outgrowth and synapse formation [[97], [98], [99], [100]]. Therefore, any alteration of BDNF levels may impair neuronal differentiation and synaptogenesis, as it has been described in AOP 13: *Chronic binding of antagonist to N-methyl*-D-*aspartate receptors (NMDARs) during brain development induces impairment of learning and memory abilities (*https://aopwiki.org/wiki/index.php/Aop:13), or AOP 54*: Inhibition of Na+/I- symporter (NIS) decreases TH synthesis leading to learning and memory deficits in children* (https://aopwiki.org/wiki/index.php/Aop:54). In these AOPs, reduced BDNF is caused by upstream inhibition of the NMDA receptor, and reduced intracellular Ca^2+^ [101] (AOP 13), or reduced levels of thyroid hormone in the developing brain (AOP 54) leading to decreased synaptogenesis and neuronal network function, and finally causing impairment in learning and memory [102]. The key event relationships (KERs) identified in these AOPs suggest that chemicals, including POPs, that downregulate BDNF protein levels, may potentially contribute to impairment of learning and memory in children through mechanisms described in these two AOPs. In our *in vitro* study, a short term (3 days) treatment with all 29 congeners (*PerF + Br + Cl*) did not alter BDNF levels (Fig. 3A, C). After 14 days, exposure to *PerF only* and *Br only* mixtures modestly decreased BDNF levels (Fig. 6A), although these effects were not associated with a decrease of synapse number or neurite length. Interestingly, decrease of BDNF (at levels comparable upon exposure to *PerF only* and *Br only*) was also observed upon treatment with the mixture containing both *PerF + Br*, and under these conditions also a remarkable decrease of synapses was recorded, supporting KER 448 (i.e., BDNF reduction leads to decreased synaptogenesis), as described in AOPs 13 and 54 in AOP-Wiki [103].

After longer treatment (28 days), no significant changes of BDNF levels could be observed under most treatment conditions, except for *PerF + Br*, *Br + Cl*, and the total mixture (*PerF + Br + Cl*) showing upregulation of BDNF levels, and suggesting that *Br* compounds may drive BDNF upregulation in combination with other congeners after prolonged exposure. It is conceivable that any alterations, i.e., increase or decrease of synaptogenesis, neurite length and BDNF levels, may in the long term (> 28 days) lead to compromised neuronal differentiation, possibly resulting in impaired neuronal network formation and function. While this was not directly verified in the present study, future investigations are warranted to prove possible perturbations of neuronal network formation and function occurring upon prolonged exposure to POP mixtures in such *in vitro* human system.

### 4.3 POP mixtures effects on AhR gene expression

AhR gene expression has been found progressively upregulated in our cell culture model (Fig. 1D), which is line with previous studies pointing at AhR as an important regulator of neuronal differentiation [[41], [42], [43], [44], [45]]. Three-day acute treatment with all 29 compounds (*PerF + Br + Cl)* at lower concentrations (0.5x and 1x), caused a decrease of AhR gene expression, supporting our previous study showing inhibition of AhR transactivation in three transgenic cell lines upon treatment with the same POP mixture [39]. On the other hand, AhR activation has been proposed as (one of) the etiological mechanism(s) underlying POP effects, with several POPs (e.g., polycyclic aromatic hydrocarbons and dioxins) acting as AhR ligands and activators of AhR signaling [47].

Apart from transactivation of AhR as a potential driver of POP-mediated DNT, other direct and indirect mechanisms have been involved, such as changes in neurotransmitters (dopamine or serotonin), alterations of intracellular phosphokinase C signaling and Ca^2+^ homeostasis, and alterations of thyroid hormone balance, as summarized by Kodavanti [104]. The lack of increase in AhR expression after acute treatment in NSCs (at 3 DIV), and the tendency (not statistically significant) towards an increase of AhR expression observed upon repeated dose treatments with POP mixtures (especially *Cl* compounds after 14-day treatment) suggest induction of AhR-mediated toxicity of POPs occurring at later stages of neuronal and glial cell differentiation. Along this line, exposure to *PerF + Br + Cl* (at 1x concentration) did not inhibit the observed increase of AhR gene expression occurring during differentiation (Supplementary Fig. 1B). Future studies will help elucidate the toxicodynamics of AhR signalling regulation upon treatment with POP mixtures in this test system.

### 4.4 Possible implications of the observed effects in neurodevelopmental disorders

Alterations of BDNF level have been implicated in a large range of neurological disorders. For instance in a recent study in newborns, lower blood level of BDNF was significantly associated with increased odds of developing ASD, suggesting that lower BDNF levels in newborns may contribute to the etiology of ASD [105]. Previous studies show that mice heterozygous for targeted disruption of BDNF, NT4/5, NT3, TrkC and TrkA exhibited hyperactive behavior compared with wild-type littermates [106]. Similarly, BDNF knock out (KO) mice exhibited a phenotype that displayed hyperactive behaviors [107], while another study suggested that this effect was sex-specific, since male BDNF conditional KOs exhibited hyperactivity, whereas female BDNF KOs exhibited normal locomotor activity [108]. This is in line with a systematic review, concluding that peripheral BDNF levels were significantly higher in males with ADHD compared with controls, whereas there was no difference in BDNF levels between ADHD female patients and control group [109]. Schizophrenia patients generally have lower than normal levels of BDNF, which is thought to contribute to dysfunctional neural networks with altered neuroplasticity and synaptic formation/function, which makes the neural network more vulnerable to disturbances [[110], [111], [112]]. Interestingly, BDNF decreased significantly in hippocampus in rodents after prenatal exposure to brominated flame retardants, like BDE-209 [113], BDE-99 [114] and BDE-47 [115]. Also, PFOS decreased BDNF levels in SH-SY5Y cells [116], and in rats on PND 35, while an increase was observed at PND 7 [117].

Multiple studies have revealed that in ASD, mutations in certain genes (e.g., NRXN, NLGN, SHANK, or MECP2) converge on common cellular pathways involved in impairment of synaptogenesis [118], abnormal neurite formation between adjacent cells, causing impairment of the brain ability to integrate the information coming from various brain regions [119]. Here, we did observe an increase in neurite length upon exposure to POPs mixtures containing *Cl* compounds, especially after 14-day treatment (Fig. 7).

Furthermore, it was found that ASD synaptopathology is accompanied by changes in neurite morphology (shorter and less branched) [120,121], and increased number of neurons in the prefrontal cortex (approximately by 67 %) compared with healthy control children [122]. Moreover, about 80 % of the genes that are considered to be high-risk for ASD play an important role in early neurodevelopmental differentiation and functions, in particular neurite outgrowth and synapse formation [123]. Interestingly, also in our studies, after 3 day exposure to all 29 congeners (*PerF + Br + Cl*), and 28 day exposure to *PerF + Br + Cl*, *PerF + Cl*, *Br + Cl*, *PerF only* and *Br only* at concentrations found in human blood levels, we observed a tendency towards an increased number of neurons (MAP2+) (Fig. 7). Additionally, an increase in the amount of proliferating NSCs (nestin+/Ki67+) was observed (Fig. 7), and according to the Loewe non-interaction model, after 14 days all tested pairwise combinations of POPs classes affected the percentage of nestin+/Ki67+ cells beyond the null model, suggesting combined/potentiated effects (Fig. 8).

This indicates that POPs at concentrations relevant to human exposure (or lower) may either stimulate NSC proliferation, or impair NSC capability to undergo physiological apoptosis, resulting in augmented percentage of neurons. Interestingly, the observed increase of neuronal cell proportion over control levels to a certain extent reproduces autism-like features observed in the brain of ASD children [122]. It would be interesting to investigate whether this increased proportion of proliferating nestin+ cells would differentiate into mature neurons upon even longer exposure to POPs (> 28 days) and if so, towards which neuronal subtype.

With regard to astrocytes, their percentage was only slightly modified (i.e., either decreased or increased depending on mixture types) upon exposure to POPs for 3, 14 and 28 days (Fig. 7). However, even a minor alteration in astrocyte population could compromise neuronal survival/function, since astrocytes play an important supportive role in defence mechanisms, including antioxidant response, as well as brain bioenergetic coupling [124].

This study supports that human NSC-derived neuronal/glial cultures are relevant *in vitro* models, suitable to test toxic effects of environmental chemicals in support of associations between chemical exposure and neurodevelopmental deficits observed in epidemiological studies. The use of models relevant to human biology, in combination with a battery of *in vitro* assays anchored to common key events identified in the DNT AOP network, represents a reliable mechanistic approach to study DNT effects induced not only by single chemicals, but, as shown in this study, it also provides new insights into toxicity evaluation of combined exposures to multiple chemicals at a time, which is very challenging to handle in both regulatory and epidemiological studies. Combining the use of an *in vitro* human relevant model with mathematical modelling, such as the Loewe non-interaction model, may be a suitable approach to enable prediction of reversibility (due to induction of compensatory mechanisms) or irreversibility of elicited effects especially after long term exposure to multiple chemicals.

It has to be stressed that the present study is based on levels of POPs found in human blood, but it is likely that considerable variations in exposure-concentrations may occur during different stages of brain development.

Another aspect to take into account is that classical monotonic dose-response trends could not be observed for most of the endpoints and treatment conditions analysed in this study (especially after 14 and 28-day treatment, Fig. 5, Fig. 6). This may (also) be linked to the design of the experimental setting, i.e., testing over prolonged periods of time fairly complex mixtures of POPs at low concentrations in a dynamic test system (i.e., NSCs undergoing neuronal and glial cell differentiation during 28 DIV, which involves changes in the proportion of different cell subpopulations over time, as shown in Fig. 1). Compensatory and adaptive mechanisms, especially in long-term treatments, may also play an important role [[125], [126], [127]]. However, it is important to learn how to tackle such dynamic *in vitro* systems where population of cells and their state of development/differentiation change considerably during long-term experiments, starting from NSC proliferation until synaptogenesis takes place, reflecting rapid brain development processes occurring *in vivo*.

Indeed, the more remarkable effects observed after 3-day (acute) treatment can also be explained from a biological standpoint. At a very early stage of differentiation, NSCs are still proliferating, with minimal (or null) defence mechanisms (e.g., lower AhR gene expression (Fig. 1D), and lower Nrf2/ARE signaling pathway activation [68] than in differentiated cell culture), and almost no glial cells are present, which play an important role in neuronal protection in response to chemical-induced toxicity. In line with this, we have previously reported that human pluripotent stem cells at an early stage of neuronal differentiation are more sensitive to chemical treatments (e.g., methylmercury) than their mature/differentiated counterparts [128].

Assessment of individual congeners’ effects and their intra- and extra-cellular distribution by analysis of biokinetics and their possible bioactivation may also help elucidate some of the possible reasons behind the relatively high variability in POP mixture effects observed in these results. Additionally, POPs are lipophilic and tend to accumulate in the cells; therefore, biokinetics study will be performed in the future to determine the levels of intracellular POP accumulation occurring after prolonged treatments.

## 5 Conclusions

Our results show that exposure to POP mixtures at concentrations found in human blood may cause alterations of synaptogenesis, neurite outgrowth, synapse formation and BDNF levels in a human-based mixed culture of neurons and astrocytes undergoing differentiation. Amongst the selected *in vitro* DNT endpoints, synaptogenesis and neurite outgrowth were the most sensitive to POP mixture-induced DNT effects, as indicated by mathematical modelling. While our findings do not always support key event relationships described in the DNT AOP network (e.g., KER 448), any perturbation (up or down) of these neurodevelopmental processes may lead to impairment of brain development. The obtained results contribute to better understanding of the possible link between human exposure to POP mixtures and neurodevelopmental disorders, including ADHD, ASD and impairment of learning and memory in children, whose prevalence has dramatically increased over the last decades.

## Conflict of Interest

The authors declare no conflict of interest.

## Funding

The current work was supported by the Norwegian Research Council, project 213076/H10 and project 204361/H10.

## Declaration of Competing Interest

The authors report no declarations of interest.
