# Supplementary material for: Exposure to human relevant mixtures of halogenated persistent organic pollutants (POPs) alters neurodevelopmental processes in human neural stem cells undergoing differentiation
Source: Reprod Toxicol. 2021 Mar;100:17–34. doi: 10.1016/j.reprotox.2020.12.013 (PMC7992035; doi:10.1016/j.reprotox.2020.12.013)
Supplement: Supplementary file 1 [file mmc1.docx]

| **Supplementary Table 1.** Sources, purities and CAS numbers of chemicals used in the study. | | | | |
| --- | --- | --- | --- | --- |
| **Chemical** | **Purity (%)^a^** | **Source** | **Cat. No.** | **CAS No.** |
| PCB 28 | 99 (99.5) | Chiron AS, Trondheim, Norway | RE-REPET195N | 7012-37-5 |
| PCB 52 | 98.5 (99.5) | Chiron AS, Trondheim, Norway | RE-REPET196N | 35693-99-3 |
| PCB 101 | 99.5 (99.5) | Chiron AS, Trondheim, Norway | RE-REPET197N | 37680-73-2 |
| PCB 118 | (99.5) | Chiron AS, Trondheim, Norway | RE-REPCB1043N | 31508-00-6 |
| PCB 138 | 99 (99) | Chiron AS, Trondheim, Norway | RE-REPET198N | 35065-28-2 |
| PCB 153 | 98.5 (99.5) | Chiron AS, Trondheim, Norway | RE-REPET199N | 35065-27-1 |
| PCB 180 | 98 (99) | Chiron AS, Trondheim, Norway | RE-REPET200N | 35065-29-3 |
| *p,p’*-DDE | (99.5) | Chiron AS, Trondheim, Norway | RE-REPET102N | 72-55-9 |
| HCB | (>99.5) | Chiron AS, Trondheim, Norway | RE-REPET145N | 118-74-1 |
| α-Chlordane | (>99.5) | Chiron AS, Trondheim, Norway | C3334.10-100-CY | 5103-71-9 |
| Oxychlordane | (>99) | Chiron AS, Trondheim, Norway | C3335.10-100-CY | 27304-13-8 |
| *trans*-Nonachlor | (>99.5) | Chiron AS, Trondheim, Norway | RE-REPET171N | 39765-80-5 |
| α-HCH | (>99.5) | Chiron AS, Trondheim, Norway | RE-REPET140N | 319-84-6 |
| β-HCH | (>99.5) | Chiron AS, Trondheim, Norway | RE-REPET141N | 319-85-7 |
| γ-HCH (Lindane) | (>99.5) | Chiron AS, Trondheim, Norway | RE-REPET148N | 58-89-9 |
| Dieldrin | (>99) | Chiron AS, Trondheim, Norway | RE-REPET124N | 60-57-1 |
| BDE-47 | 98.5 (99.5) | Chiron AS, Trondheim, Norway | RE-REPBDE0047N | 5436-43-1 |
| BDE-99 | 99.5 (99.5) | Chiron AS, Trondheim, Norway | RE-REPBDE0099N | 60348-60-9 |
| BDE-100 | 99.5 (99.5) | Chiron AS, Trondheim, Norway | RE-REPBDE0100N | 189084-64-8 |
| BDE-153 | 99 (99.5) | Chiron AS, Trondheim, Norway | RE-REPBDE0153N | 68631-49-2 |
| BDE-154 | 99 (99) | Chiron AS, Trondheim, Norway | RE-REPBDE0154N | 207122-15-4 |
| BDE-209 | 99 (99.5) | Chiron AS, Trondheim, Norway | RE-REPBDE0209N | 1163-19-5 |
| HBCD | ≥95 | Sigma-Alrich, St. Louis, MO, USA | 144762 | 3194-55-6 |
| PFHxS (potassium salt) | >98 | Santa Cruz Biotechnology, Inc., Dallas, TX, USA | sc-237289 | 3871-99-6 |
| PFOS (potassium salt) | ≥98 | Sigma-Alrich, St. Louis, MO, USA | 77282 | [2795-39-3](https://www.sigmaaldrich.com/catalog/search?term=2795-39-3&interface=CAS%20No.&N=0&mode=partialmax&lang=en&region=NO&focus=product) |
| PFOA | 96 | Sigma-Alrich, St. Louis, MO, USA | 33824 | 335-67-1 |
| PFNA | 97 | Sigma-Alrich, St. Louis, MO, USA | 394459 | 375-95-1 |
| PFDA | 98 | Sigma-Alrich, St. Louis, MO, USA | 177741 | 335-76-2 |
| PFUnDA | 95 | Sigma-Alrich, St. Louis, MO, USA | 446777 | [2058-94-8](https://www.sigmaaldrich.com/catalog/search?term=2058-94-8&interface=CAS%20No.&lang=en&region=US&focus=product) |
| ^a^ Chemical purity percentages are as determined by HPLC or in GC-MS (in parentheses)  Adapted from McComb et al. (2019) with modifications.  J. McComb, I.G. Mills, M. Muller, H.F. Berntsen, K.E. Zimmer, E. Ropstad, S. Verhaegen, L. Connolly. Human blood-based exposure levels of persistent organic pollutant (POP) mixtures antagonise androgen receptor transactivation and translocation. Environ Int. 132 (2019) 105083. doi: 10.1016/j.envint.2019.105083. | | | | |

| **Supplementary Table 2. Quantification of NSCs in control culture after 1, 14 and 28 days in vitro (DIV).** Cell percentages were quantified using the ArrayScan™ XTI High Content Platform (Cellomics) and the ArrayScan 'Neuronal Profiling V4.1' BioApplication (4 independent experiments, 6 internal replicates). | | | | | | | | | |
| --- | --- | --- | --- | --- | --- | --- | --- | --- | --- |
|  | | **Exp1** | | **Exp2** | | **Exp3** | | **Exp4** | |
|  | **DIV** | **Mean** | **SD** | **Mean** | **SD** | **Mean** | **SD** | **Mean** | **SD** |
| Nestin | 1 | 62.69 | 10.35 | 60.50 | 7.58 | 57.48 | 11.97 | 57.33 | 6.74 |
|  | 14 | 46.94 | 16.28 | 52.33 | 15.14 | 51.75 | 20.35 | 65.61 | 9.69 |
|  | 28 | 20.03 | 3.91 | 30.67 | 11.78 | 29.50 | 5.89 | 36.83 | 7.49 |
