# Supplementary material for: Exposure to human relevant mixtures of halogenated persistent organic pollutants (POPs) alters neurodevelopmental processes in human neural stem cells undergoing differentiation
Source: Reprod Toxicol. 2021 Mar;100:17–34. doi: 10.1016/j.reprotox.2020.12.013 (PMC7992035; doi:10.1016/j.reprotox.2020.12.013)
Supplement: Supplementary file 2 [file mmc2.docx]

**Supplementary Figure legend**

**Supplementary Figure 1.** **AhR gene expression upon POP mixtures treatment.** (A) Gene expression analysis of AhR gene upon 14 day- (red bars) and 28 day-treatment (black bars) with all seven POP mixtures at 1x concentrations (blood levels) (AhR gene expression was normalized to βactin and GAPDH and calibrated on the respective solvent control cells). (B) AhR gene expression comparing solvent control culture (green bars) and cells treated with *PerF + Br + Cl* mixture at 1x concentration (red bars), after 3, 14 and 28 DIV (paired comparisons between control culture and treated cells were analyzed by one-tailed paired t-test; AhR gene expression was normalized to Bactin and GAPDH and calibrated on solvent control cells at 3 DIV). Mean ± S.E.M. of 3 biological replicates.
