# Supplementary figures and images for: Exposure to human relevant mixtures of halogenated persistent organic pollutants (POPs) alters neurodevelopmental processes in human neural stem cells undergoing differentiation
Source: Reprod Toxicol. 2021 Mar;100:17–34. doi: 10.1016/j.reprotox.2020.12.013 (PMC7992035; doi:10.1016/j.reprotox.2020.12.013)

**A**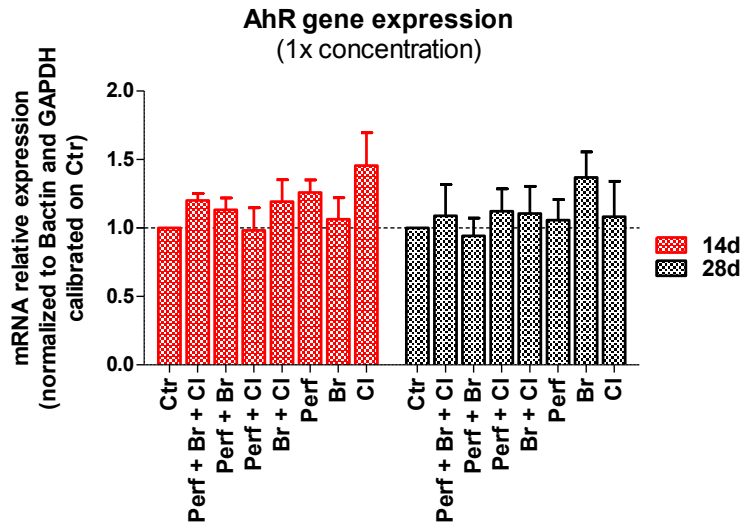**B**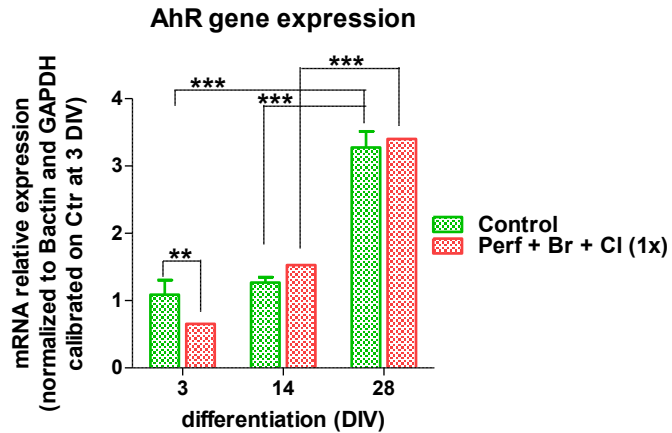

Supplement: Supplementary file 3 [file mmc3.pdf]
